# The Angle System of Regulation and Retention of the Teeth

**Published:** 1893-03

**Authors:** 


THE ANGLE SYSTEM OF REGULATION AND RETENTION OF THE TEETH. (Third edition, revised and enlarged.) By Edward H. Angle, D.D.S., former Professor of Histology and Orthodontia in the Dental Department of the University of Minnesota. Published by the Wilmington Dental Manufacturing Co., Philadelphia. 75 cents.

In this little book we find a great amount of valuable information. Dr. Angle has from time to time given the profession numerous suggestions in orthdontia, treatment of fractures of the maxillæ, etc., which are always pre-eminently practical, issuing, evidently, from a practical mind.

We have long ago recognized the superiority of this system of regulation over many of the clumsy and often unscientific appliances in common use, and would recommend a careful perusal of Dr. Angle's work. W.

